# Asthma and hypertension: the role of airway inflammation

**DOI:** 10.3389/fmed.2024.1451625

**Published:** 2024-10-10

**Authors:** Dina Visca, Francesco Ardesi, Martina Zappa, Patrizia Pignatti, Sarah Grossi, Marco Vanetti, Giovanni Battista Migliori, Rosella Centis, Fabio Angeli, Antonio Spanevello

**Affiliations:** ^1^Department of Pulmonary Rehabilitation, Istituti Clinici Scientifici Maugeri IRCCS, Tradate, Italy; ^2^Department of Medicine and Surgery, University of Insubria, Varese, Italy; ^3^Allergy and Immunology Unit, Istituti Clinici Scientifici Maugeri IRCCS, Pavia, Italy; ^4^Servizio di Epidemiologia Clinica delle Malattie Respiratorie, Istituti Clinici Scientifici Maugeri IRCCS, Tradate, Italy; ^5^Department of Medicine and Technological Innovation (DiMIT), University of Insubria, Varese, Italy; ^6^Department of Medicine and Cardiopulmonary Rehabilitation, Istituti Clinici Scientifici Maugeri IRCCS, Tradate, Italy

**Keywords:** asthma, chronic diseases, comorbidities, arterial hypertension, airway inflammation, induced sputum

## Abstract

**Introduction:**

Asthma is a chronic inflammatory respiratory disease often associated with comorbidities. Among cardiovascular comorbidities, arterial hypertension seems to create an additional health burden in asthmatics. However, evidence on this relationship is lacking.

**Objective:**

Our study aims to evaluate the characteristics of hypertensive asthmatics, focusing on the role of inflammation as a possible link between these diseases.

**Methods:**

We conducted a monocentric retrospective analysis consecutively including asthmatics who underwent induced sputum (IS) at our asthma referral center. Patients were divided in two groups according to presence or absence of history of hypertension. Clinical, functional, and inflammatory (airway and systemic) data were collected.

**Results:**

Data on two hundred and sixty asthmatic patients were analyzed. Seventy-nine (30.4%) of them had a diagnosis of hypertension requiring a specific pharmacological treatment. Asthmatics with hypertension were more frequently male (*p* = 0.047), older (*p* < 0.001), and with higher body max index (BMI) (p < 0.001) when compared to normotensive patients. No difference concerning asthma control, severity and pharmacological treatment was observed between the two groups (all *p* > 0.05); distribution of comorbidities and lung function impairment (forced expiratory volume in the first second (FEV1) and forced vital capacity (FVC); all *p* < 0.05) were statistically different between groups. Mixed granulocytic airway inflammation was prevalent in the hypertensive asthmatics (*p* = 0.014). Interestingly, a multivariable analysis revealed that age ≥ 65 years and an increased percentage of sputum neutrophils (≥61%) were independent predictors of hypertensive status (*p* < 0.001).

**Conclusion:**

Our data suggest that neutrophilic airway inflammation (as evaluated by induced sputum) is strictly associated with hypertension. In clinical practice, phenotyping asthmatic patients with comorbidities like hypertension could be useful also from a therapeutic point of view. Additional studies are mandatory to further elucidate the role of neutrophilic airway inflammation in asthma with cardiovascular diseases.

## Introduction

Asthma is a heterogeneous respiratory disease usually characterized by chronic airway inflammation and bronchial hyperactivity (1). Asthma is a global health issue that affects around 300 million people of all ages worldwide (2). It is well known that asthma is associated with different comorbidities (3, 4) such as chronic rhinosinusitis with nasal polyps (CRSwNP) (5, 6), gastro-esophageal reflux disease (GERD) (7), obesity (8) and arterial hypertension (9). Essential hypertension is one of the leading chronic cardiovascular diseases affecting 1.3 billion people and 33% of adults between 30 and 79 years of age worldwide (10).

Population-based studies reported that asthmatic patients are over 40% more likely to have hypertension than patients without bronchial asthma (11–13). Moreover, it has been demonstrated that arterial hypertension in asthma patients is associated with enhanced disease morbidity and worse disease control (14, 15). Although these two diseases may be linked, most of the pathophysiological features of their relationship are still unclear.

A key role in the development of both asthma and hypertension is therefore played by inflammation.

Inflammatory process in asthma is usually classified into two main pathways: Type 2 (T2) high and T2-low inflammation. The former is characterized by predominant eosinophilic airway inflammation related to a T2 immune response. On the other hand, T2-low asthma usually has neutrophilic or paucigranulocytic airway inflammation and is considered to be driven by nonallergic/non-eosinophilic response (16). The latter pathway appears to have greater links to inflammation due to hypertension (17). Indeed, these two diseases may share some immune mechanisms such as increased expression of interferon-*γ*, macrophage activation and high serum level of interleukin-6 (IL-6) (18, 19).

Despite different studies have investigated this inflammatory link by analyzing the role of neutrophils and different cytokines in blood, the role of bronchial inflammation has been poorly investigated and, to the best of our knowledge, the induced sputum in bronchial asthma with hypertension was never investigated.

Phenotyping asthma patients, especially from an inflammatory point of view, has become crucial to better understand the disease and optimize treatment. In clinical practice, blood eosinophils, fractional exhaled nitric oxide (FeNO), and serum total IgE are the most used biomarkers to discriminate between T2-high and T2-low asthma (1). However, induced sputum (IS) is the gold standard to non-invasively assess central airway inflammation.

The aim of this study is to compare clinical, functional, and inflammatory characteristics of asthmatic patients with and without hypertension and investigate whether airway inflammation may help to identify a subgroup of patients at high risk of having hypertension.

## Methods

### Study design

This retrospective observational study was performed at an Italian referral asthma center (Istituti Clinici Scientifici (ICS) Maugeri, IRCCS, Tradate, Italy).

Demographics, clinical and biological data, on asthmatic (1) outpatients with or without hypertension (20) attending the clinic between 2017 and 2023, were collected in a de-identified database.

The study was performed according to the Declaration of Helsinki and approved by the Internal Review Board of ICS Maugeri of Tradate Institute (Identifier: p3/16). All participants gave written informed consent for their participation in the research.

All stable asthmatic patients with at least one IS examination were consecutively included in the analysis. The history of other acute cardiovascular diseases and asthma exacerbations in the month prior to the visit were considered as exclusion criteria. Patients on biological treatment for severe asthma were excluded. Asthma with hypertension (AwH) patients were on stable treatment for hypertension according to the ESC/ESH guidelines (20).

### Measurements

We reviewed patients’ electronic medical records to collect the following data: baseline characteristics (age, sex, body mass index (BMI), smoking history), atopy, symptoms control (Asthma Control Questionnaire-6 (ACQ-6)) (21–23), episodes of acute exacerbations, pharmacological treatment, comorbidities and healthcare utilization.

Atopy, according to the World Allergy Organization definition, was assessed by prick tests and/or specific IgE in serum for the most common inhalant allergens. Exacerbations were defined by a course of systemic corticosteroids (OCS) for at least 3 days in case of asthma symptoms worsening. Healthcare utilization was defined as admission to Emergency Department or additional visits due to asthma in the previous 12 months.

Lung functional evaluation was performed through spirometry test (MIR MiniSpir, MIR, Italy) according to the American Thoracic Society (ATS)/European Respiratory Society (ERS) guidelines (24, 25) and the Global Lung Function Initiative (GLI) reference equations adopted immediately for spirometry standardized lung function values (26).

Both FEV1 and FVC were recorded; these measurements were expressed as either absolute value (liters (L)) or percentage of their predicted value (% predicted). The FEV1/FVC value was recorded as a % ratio. To ensure spirometry reproducibility, at least three maneuvers were taken, and lung volumes variability were assessed during each spirometry test. The bronchodilator test was also conducted to determine the reversibility of airflow limitation after administration of an inhaler short-acting bronchodilator drug (salbutamol 400 μg) as a part of lung function tests.

Inflammatory data were also collected: blood and sputum samples were collected on the same day. Blood eosinophils and neutrophils (expressed as cells/mm^3^ and as % of white blood cells) were measured as part of the complete blood and differential cell count.

The IS test was performed according to ATS/ERS guidelines to study bronchial inflammation (27). Inflammatory patterns were defined as follows: eosinophilic when eosinophils ≥3% and neutrophils <61%, neutrophilic when neutrophils ≥61% and eosinophils <3%, mixed granulocytic when eosinophils ≥3% and neutrophils ≥61% and paucigranulocytic when eosinophils <3% and neutrophils <61% (28–30).

### Statistical analysis

An *ad hoc* electronic form was created to collect all study variables. Qualitative variables were described with absolute and relative (percentages) frequencies, whereas quantitative variables were shown based on their normal or non-normal distribution as mean (standard deviations: SD) or median (interquartile ranges: IQR), respectively. The Kolmogorov–Smirnov test was used to assess the normality of distribution for all variables. Comparisons between the two groups for qualitative and quantitative variables were assessed with the χ2 or Fisher’s exact test when appropriate, while Student T-test and Mann–Whitney were used in case of parametric or nonparametric distribution.

Predictors of hypertension were evaluated using univariate and multivariable logistic regression analysis (reporting odds ratio [OR] and 95% CI). A *p*-value <0.05 was considered statistically significant. Stata 17 statistical software (StatsCorp, Texas, USA) was used for every statistical computation.

## Results

Two hundred and sixty asthmatic patients were consecutively included in the study. The study population was subsequently divided into two groups based on the presence or absence of history of hypertension: 79 patients (30.4%) with diagnosis of hypertension requiring a specific pharmacological treatment were included in the asthma with hypertension group (AwH) and 181 (69.6%) in the asthma no hypertension (AnH) group.

The clinical and demographic characteristics of all patients are listed in Table 1.

**Table 1:** Baseline characteristics.

Variables		AnH = 181	AwH = 79	*p*-value
Male, *n* (%)		70 (38.7)	41 (51.9)	**0.047**
Age, years		56 (45–63)	64.5 (56–72)	**<0.001**
BMI, Kg/m^2^		24 (22.0–27.3)	27 (24.5–32.0)	**<0.001**
Smoking habit, *n* (%)	Never-smoker	114 (62.9)	42 (53.2)	0.184
	Current smoker	10 (5.6)	6 (7.6)	
	Former smoker	57 (31.5)	31 (39.2)	
Age Asthma Onset, median (IQR)		42 (23.3–55.0)	55 (45.0–66.0)	**<0.001**
Atopy, *n* (%)		113 (62.4)	41 (51.9)	0.112
ICS, *n* (%)		163 (90.0)	71 (89.9)	0.964
LTRA, *n* (%)		59 (32.6)	32 (40.5)	0.230
mOCS, *n* (%)		32 (17.8)	11 (13.9)	0.453
Step GINA, *n* (%)	1	16 (8.8)	7 (8.8)	0.947
	2	4 (2.2)	2 (2.5)	
	3	27 (14.9)	12 (15.2)	
	4	51 (28.2)	19 (24.1)	
	5	83 (45.9)	39 (49.4)	
Beclomethasone HFA equivalent, median (IQR)		400 (320–800)	440 (320–800)	0.520
MART treatment, *n* (%)		92 (50.8)	40 (50.6)	0.977
ACQ, median (IQR)		0.5 (0.0–1.50)	0.33 (0.0–1.00)	0.081
Exacerbations (previous year), *n* (%)		83 (45.9)	33 (41.8)	0.518
Mild Exacerbations, median (IQR)		0 (0–2)	0 (0–2)	0.929
Severe Exacerbations, median (IQR)		0 (0–0)	0 (0–0)	0.689
Healthcare utilization, *n* (%)		79 (43.6)	25 (31.6)	0.064

Data are presented as number (*n*) and percentage (%) for dichotomous values or median (IQR) for skewed variables. AnH, asthma no hypertension; AwH, asthma with hypertension; IQR, inter-quartile range; BMI, body mass index; ICS, inhaled corticosteroids; LTRA, Leukotriene Receptor Antagonists; mOCS, maintenance oral corticosteroids; GINA, Global Initiative for Asthma; HFA, hydrofluoroalkane; MART, Maintenance and Reliever Therapy; ACQ, Asthma Control Questionnaire. Bold *p*-value <0.05.

No differences concerning asthma severity (according to GINA document) (1) and asthma therapy were observed between the two groups.

There were no statistically significant differences in smoking habits or asthma control (number of exacerbations per year, ACQ-6 scores, and healthcare utilizations in the previous year) between AnH and AwH. The majority of AwH patients were male (51.9% vs. 38.7% in AnH, *p* = 0.047) older (median (IQR): 64.5 (56–72) vs. 56 (46–63) in AnH *p* < 0.001), had higher BMI (median (IQR) 27 (24.5–32) vs. 24 (22.0–27.3) in AnH *p* < 0.001).

**Figure 1:**
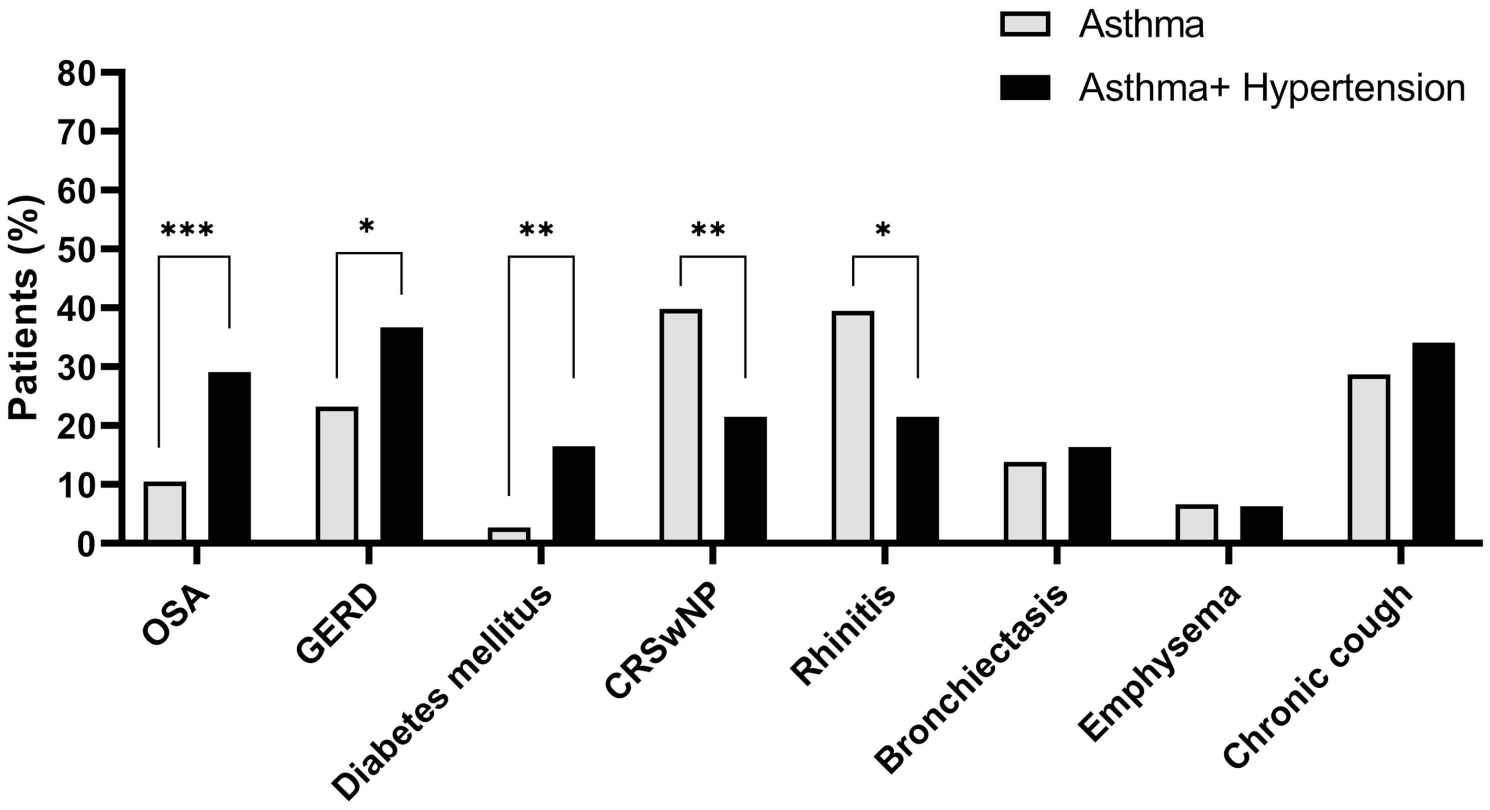
Comorbidities in asthmatic patients with or without hypertension. Data are presented as percentage (%). OSA, Obstructive Sleep Apnea; GERD, gastroesophageal reflux disease; CRSwNP, chronic rhinosinusitis with nasal polyps. *p-value <0.05; **p < 0.01; ***p < 0.001.

Two hundred and twenty-six patients (86.2%) had at least one comorbidity with different distribution in the two groups. The majority of the AwH group had less CRSwNP (21.5% vs. 39.8% in AnH *p* = 0.004), rhinitis (21.5% vs. 39.5% in AnH *p* = 0.022) and more GERD (36.7% vs. 23.2% in AnH *p* = 0.025), obstructive sleep apnoea (OSA) (29.11% vs. 10.5% in AnH *p* < 0.001) and diabetes mellitus (16.5% vs. 2.7% in AnH *p* = 0.001) (Figure 1).

The lung function evaluation documented differences between the two groups as shown in Table 2. AwH patients had a worse lung function impairment compared to AnH ones in terms of FEV1 (L) (median (IQR) 2.08 (1.73–2.69) vs. 2.52 (1.94–3.12), *p* < 0.001) and FVC (L) (3.08 (0.97) vs. 3.59 (1.11), *p* < 0.001) but not for the FEV1/FVC ratio (median (IQR) 72.7 (66.0–79.0) 73.0 (65.4–79.8), *p* = 0.929). These data are confirmed also after the bronchodilation test (median (IQR): FEV1 (L) post 2.17 (1.74–2.70) vs. 2.72 (2.17–3.23), *p* < 0.001); FVC (L) post 2.93 (2.41–3.73) vs. 3.50 (2.91–4.19), *p* < 0.001; FEV1/FVC post 74.00 (68.00–82.00) vs. 76.00 (68.00–82.03), *p* = 0.606. However, and as aforementioned, BMI showed higher mean values in AwH when compared with AnH, suggesting a potential role of restrictive airway limitation due to obesity (Table 1).

**Table 2:** Clinical functional and inflammatory characteristics.

Variables		AnH = 181	AwH = 79	*p*-value
FEV1, L		2.52 (1.94–3.12)	2.08 (1.73–2.69)	**<0.001**
FEV_1_, % predicted		91.5 (79.0–105.0)	87.0 (68.0–106.0)	0.351
FVC, L		3.59 (1.11)	3.08 (0.97)	**0.001**
FVC, % predicted		101.5 (91–116.0)	94 (82–115.0)	**0.036**
FEV_1_/FVC, %		73.0 (65.4–79.8)	72.7 (66.0–79.0)	0.929
FEV1 post BD, L		2.72 (2.17–3.23)	2.17 (1.74–2.70)	**<0.001**
FVC post BD, L		3.50 (2.91–4.19)	2.93 (2.41–3.73)	**<0.001**
FEV1/FVC post BD, %		76.00 (68.00–82.03)	74.00 (68.00–82.00)	0.606
Blood Leucocytes,		6.69 (5.72–7.86)	6.98 (5.81–8.42)	0.149
Blood Neutrophils, %		53.40 (8.57)	54.20 (8.22)	0.529
Blood Lymphocytes, %		31.90 (27.30–37.65)	29.50 (25.68–35.90)	0.084
Blood Eosinophils, %		4.00 (2.05–7.20)	3.95 (1.98–6.60)	0.795
Blood Eosinophils, ucl		262.40 (139.10–533.04)	269.62 (130.24–473.72)	0.804
Blood Neutrophils, ucl		3542.40 (2862.0–4261.78)	3730.10 (3232.10–4618.90)	0.078
Blood Lymphocytes, ucl		2097.03 (1793.45–2490.68)	2084.10 (1706.37–2601.27)	0.789
NLR, median (IQR)		1.67 (1.27–2.16)	1.84 (1.40–2.27)	0.112
Total IgE, U/L		199.00 (91.90–578.00)	177.0 (45.30–437.00)	0.696
Cells x10^4^/ml IS,		148.50 (64.00–267.00)	140.00 (75.00–285.00)	0.856
IS Macrophages %, median (IQR)		17.70 (7.93–29.70)	15.80 (6.30–26.10)	0.203
IS Neutrophils %, median (IQR)		51.15 (20.60–74.18)	63.40 (21.90–78.70)	0.072
IS Eosinophils %, median (IQR)		6.60 (0.95–33.97)	5.60 (1.90–24.30)	0.656
IS Lymphocytes %, median (IQR)		1.00 (0.40–2.00)	1.20 (0.40–1.90)	0.681
IS Epithelial %, median (IQR)		3.30 (1.60–7.85)	3.70 (1.60–6.30)	0.718
IS Viability %, median (IQR)		82.30 (66.58–90.43)	82.00 (68.00–90.00)	0.881
IS Neutrophils ≥61%, *n* (%)		71 (39.2)	44 (55.7)	**0.014**
Inflammatory patterns *n* (%)	Neutrophilic	48 (26.5)	21 (26.6)	0.007*
	Eosinophilic	86 (47.5)	24 (30.4)	
	Mixed granulocytic	23 (12.7)	23 (29.1)	
	Paucigranulocytic	24 (13.3)	11 (13.9)	

Data are presented as number (*n*) and percentage (%) for dichotomous values or mean (± standard deviation) for quantitative variables or median (IQR) for skewed variables. AnH, asthma no hypertension; AwH, asthma with hypertension; FEV1, forced expiratory volume in the first second; IQR, inter-quartile range; FVC, forced vital capacity; BD, bronchodilator test; NLR, blood neutrophil-to-lymphocyte ratio IS, induced sputum.; *Eosinophilic pattern, *p* = 0.01; Mixed granulocytic pattern, *p* = 0.014. Bold *p*-value <0.05.

Airway inflammation was similar in the two groups in terms of cellular percentage; however, in the AwH group, the percentage of sputum neutrophils was ≥61% (cut-off to define sputum neutrophilia) (28) in 55.7% patients vs. 39.2% in the AnH group (*p* = 0.014) (Table 2). Among hypertensive patients, neutrophils count was not significantly associated with age or male sex (all *p* > 0.05). No differences were found in terms of total cell counts when comparing: patients with sputum neutrophils ≥61% vs. <61% in the entire cohort of 260 asthmatics (*p* = 0.420); patients with sputum neutrophilia in AwH group vs. AnH group (*p* = 0.299); patients with sputum neutrophils ≥61% vs. <61% in AwH group (*p* = 0.864); patients with sputum neutrophils ≥61% vs. <61% in AnH group (*p* = 0.321).

**Figure 2:**
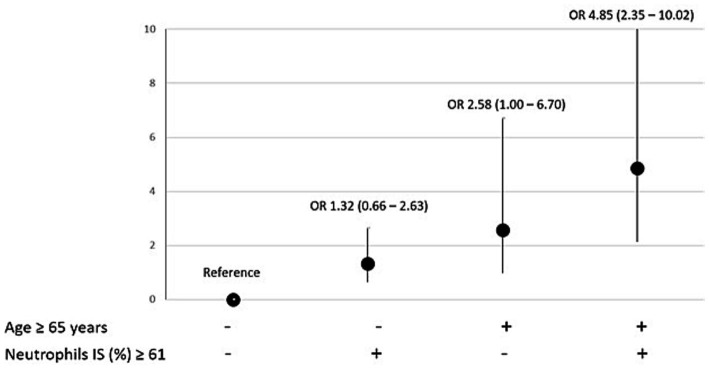
Factors associated with hypertension: effects of older age and higher neutrophils count (and their combinations) on the risk of having hypertension. Predictors of hypertension were reported as odds ratio (OR) and 95% confidence interval (CI). IS, induced sputum.

Among potential clinical and laboratory features associated with hypertension (including age, neutrophils, eosinophils, obesity, OSA, diabetes, sex, and smoking habits), only neutrophils ≥61% in induced sputum and age ≥ 65 (31) were independent predictors of hypertensive status when forced in the same multivariable model. Indeed, the coexistence of age > 65 years and neutrophils ≥61% was associated with a 5-fold increased risk of hypertension in asthmatic patients (OR: 4.85; 2.34–10.02, *p* < 0.001; Figure 2). Notably, this association was not influenced by obesity (*p* = 0.172) and eosinophils in induced sputum (*p* = 0.250).

## Discussion

The association between asthma and arterial hypertension, well documented by several studies (14, 15) is also supported and strengthened by our findings.

The prevalence of hypertension in asthmatic patients is still debated, ranging from around 20–45%, up to about 75% when actively searched (9, 12, 13, 15). An observational study by Di Raimondo et al. reported that the prevalence of hypertension is higher in asthmatic patients than controls when blood pressure is monitored (75% and up to 80.8% in severe asthma) (9).

In the present study, the prevalence of hypertension in our population is in line with previous studies being 30.4%, and as reported by Panek et al., it did not significantly increase with asthma severity (31.0, 28.4 and 32.0%, respectively, in mild, moderate, and severe asthma) (15).

Although no significant differences were found comparing asthma control, disease severity, exacerbations rate and pharmacological treatment between the AnH and the AwH group, additional studies are needed to deeply investigate the potential impact of asthma medication on the development of hypertension and vice versa. Indeed, previous studies have documented the potential role of the corticosteroid therapy (oral and inhaled) in the development of hypertension due to the mineral corticosteroid effects especially in predisposed asthmatic patients (1, 32). As far as the relationship of inhaled corticosteroids (ICS) with hypertension, Ferguson et al. suggested that lower doses of ICS may be protective while higher doses may lead to the opposite effect (33).

Our data on lung function tests show a worse lung function impairment in the AwH group in terms of FEV1 and FVC (liters), also after bronchodilator test. These results are line with previous studies (33, 34). Takase et al. reported an inverse cross-sectional association between pulmonary function measures (particularly FVC and FEV1) and blood pressure in the general population (34). Regarding asthmatics, Ferguson et al. showed that the odds of having hypertension increased with FEV1% decreasing (except for FEV1 < 60%), although the underline mechanism needs further investigation (33).

In our study, the hypertensive asthmatic group prevalently included male and older patients with higher BMI. These findings confirm the association between obesity, age, and cardiovascular risk also in asthma patients, suggesting that asthma and hypertension may share common risk factors. Both overweight and older age have been documented in different studies to be risk factors for arterial stiffening that accelerates vascular aging and development of hypertension in humans, although the rationale for this remains partially unclear (35, 36). The higher prevalence of hypertension could be explained with the prevalence of obesity in adult-onset asthma, although the underline mechanism has not been clarified (37, 38).

Asthma (especially in T2 low asthma) and hypertension share some immune mechanisms (39) involving higher expression of interferon-*γ*, activation of macrophages, and IL-6 (19) resulting in metabolic dysfunction that increases the morbidity associated with both hypertension and asthma (17).

Blood and sputum neutrophils play an important role in T2 low asthma (40, 41), however, neutrophilic airway inflammation has been poorly investigated in asthma with hypertension.

In our study, no difference was found in systemic and airway inflammation (cells count and percentage) between the AnH and the AwH group. However, a different distribution of airway inflammatory patterns was found in the two groups. The proportion of mixed granulocyte pattern was higher in the AwH group whereas the eosinophilic pattern was prevalent in the AnH group. These data may be partially explained by a different prevalence of comorbidities and aging in the two study populations. In fact, a higher neutrophils count has already been associated with aging, as previously reported (42–45). Moreover, in the AwH group, most of the comorbidities evaluated are known to be associated with T2 low asthma (obesity, OSA, GERD and diabetes mellitus) (46–53), while conditions associated to T2 high asthma (atopy, rhinitis and CRSwNP) (16, 54) were more common in the AnH group (7, 55). Additional studies are needed to clarify the role of each comorbidity (obesity, OSA, GERD and diabetes mellitus) on airway inflammation and hypertension in asthmatic patients.

However, airway inflammation in asthmatics with hypertension has not been well evaluated. Arterial hypertension has already been considered a chronic sub-inflammatory condition, which in the long term is capable of inducing damage in some organs. One of the most likely mechanisms related to neutrophils in hypertension seems to be vascular injury caused by reactive oxygen species released by neutrophils. Neutrophils may invade target organs and release vasoactive neurotransmitters and proinflammatory molecules, which may eventually lead to the sub-inflammatory state in arterial hypertension to start or worsen organ dysfunction (56). Although the systemic inflammatory characteristics in hypertension have been previously described, to the best of our knowledge bronchial inflammation in asthmatic patients with hypertension has been poorly investigated with no previous evidence on induced sputum.

According to a recent retrospective study conducted by Pignatti et al., a significant prevalence of hypertension was found in asthmatic patients aged ≤65 years with mixed granulocytic pattern in the airways (57). Our results confirm that patients with a mixed granulocytes inflammation as well as a percentage of neutrophils equal or above 61% were more common in the AwH group.

Asthmatic patients with a neutrophilic pattern (sputum neutrophils ≥61%) usually have higher sputum total cell count (57). This increase is mainly due to infection/colonization of the airways while the increase of sputum neutrophils is often associated with corticosteroids treatment (58, 59).

However, in our population we did not find differences in terms of total cell count when considering neutrophilic or non-neutrophilic patients among the entire cohort, in the AnH group, and in AwH group. No differences were found in the two groups also as concerns ICS or mOCS treatment. These results suggest that neutrophilia may be due more to comorbidities, including hypertension, rather than infection. To minimize this effect, we indeed selected patients free of exacerbation and on stable disease for at least one month before the assessment as described above.

Furthermore, to deeply analyze this relationship, we decided to evaluate if the presence of neutrophils could lead to a different risk of hypertension.

To the best of our knowledge, our study is the first one to show that neutrophils percentage in induced sputum is a determinant of hypertension after adjustment for the confounding effect of age. This observation supports that T2 low inflammatory pathways may provide a pivotal link between these two diseases (46, 60).

Among the limitations of our study, we report the retrospective design from a single reference center, the lack of a control group of non-asthmatic patients with hypertension, although with a relatively large cohort of patients. However, our results suggest that, considering the high prevalence of hypertension in asthma, the clinical relevance of IS in phenotyping asthmatic patients with cardiovascular comorbidities, deserve further validation.

Indeed, additional studies are needed to better identify airway inflammation in asthmatics with hypertension in order to improve clinical practice and optimize pharmacological treatment, considering that neutrophilic airway inflammation is less responsive to the inhaled and systemic pharmacological therapy available.

Finally, our results show that a more in-depth evaluation of airway inflammation in patients with asthma allows the identification of patients at increased risk of hypertensive disease. The role of airway cellularity as a linkage with hypertension remain largely unknown and needs to be more deeply investigated, as to ensure early diagnosis and implementation of effective treatment when needed.

## Conclusion

Our data suggest that neutrophilic airway inflammation is significantly associated with the presence of hypertension. Induced sputum evaluation is the only non-invasive methodology to assess neutrophilic airway inflammation and might be used to identify hypertensive patients requiring a closer surveillance program. In clinical practice, phenotyping asthmatic patients with comorbidities like hypertension could be useful also from a therapeutic point of view. Additional studies are needed to better understand the role of neutrophilic airway inflammation in asthma with cardiovascular diseases.

## Data Availability

The raw data supporting the conclusions of this article will be made available by the authors, without undue reservation.
